# Comparative risk of uveitis with Janus kinase inhibitors versus tumor necrosis factor inhibitors in ankylosing spondylitis and psoriatic diseases: a target trial emulation study

**DOI:** 10.3389/fimmu.2025.1673970

**Published:** 2025-10-24

**Authors:** Wei-Hsuan Bai, Pei-Lun Liao, Yi-Chiao Bai, James Cheng-Chung Wei

**Affiliations:** ^1^ Institute of Medicine, Chung Shan Medical University, Taichung, Taiwan; ^2^ Center for Health Data Science, Department of Medical Research, Chung Shan Medical University Hospital, Taichung, Taiwan; ^3^ Department of Optometry, Shu-Zen Junior College of Medicine and Management, Kaohsiung, Taiwan; ^4^ Department of Allergy, Immunology, and Rheumatology, Chung Shan Medical University Hospital, Taichung, Taiwan; ^5^ Graduate Institute of Integrated Medicine, China Medical University, Taichung, Taiwan; ^6^ Office of Research and Development, Asia University, Taichung, Taiwan

**Keywords:** janus kinase inhibitors, tumor necrosis factor inhibitors, uveitis, autoimmune disease, TriNetX, cohort study

## Abstract

**Objectives:**

To compare the risk of incident uveitis among patients with axial spondyloarthritis initiating treatment with Janus kinase inhibitors (JAKi) versus tumor necrosis factor inhibitors (TNFi).

**Methods:**

We conducted an emulated target trial using real-world electronic health records from the TriNetX US Collaborative Network. Adults with ankylosing spondylitis (AS), psoriasis (PsO), or psoriatic arthritis (PsA) who newly initiated a JAKi or a TNFi between January 1, 2016, and December 31, 2023, were identified. Patients were stratified into JAKi and TNFi cohorts based on initial treatment exposure. Propensity score matching (1:1) was performed to balance baseline demographics, comorbidities, prior medication use, and laboratory values. Cox proportional hazards models were used to estimate hazard ratio (HR) and 95% confidence interval(CI) for the development of incident uveitis, with TNFi as the reference. Kaplan–Meier analysis was conducted to compare the 9-year cumulative incidence of uveitis between cohorts. The primary outcome was incident uveitis following initiation of therapy, with follow-up extending up to nine years.

**Results:**

Among 697,850 patients identified, 5,874 were included in each group after 1:1 propensity score matching. JAKi use was associated with a lower risk of incident uveitis compared with TNFi (HR = 0.630; 95% CI, 0.418–0.948). These findings remained consistent after further adjustment for comorbidities, medications, and laboratory data. Subgroup analyses showed a consistent protective association in older patients (≥ 51 years: HR = 0.43, 95% CI = 0.24–0.79), White individuals (HR = 0.59, 95% CI = 0.38–0.93), and those with elevated inflammatory markers (CRP ≥ 3 mg/L: HR = 0.50, 95% CI = 0.26–0.96; ESR ≥ 20 mm/h: HR = 0.41, 95% CI = 0.19–0.87). The reduced risk persisted regardless of concomitant use of conventional synthetic DMARDs (with csDMARDs: HR = 0.50, 95% CI = 0.28–0.92; without csDMARDs: HR = 0.56, 95% CI = 0.33–0.94).

**Conclusions:**

In this large-scale, real-world cohort study, JAKi therapy was associated with a significantly reduced risk of incident uveitis compared to TNFi therapy in patients with AS, PsO, or PsA. These findings suggest a potential role for JAKi in mitigating ocular inflammation in this population. Further prospective studies and randomized controlled trials are warranted to validate these results and inform future clinical guidelines.

## 1 Introduction

Autoimmune diseases are driven by dysregulated innate and adaptive immune responses, leading to chronic inflammation (1). Axial Spondyloarthritis (axSpA), predominantly affecting the sacroiliac joints and spine, including ankylosing spondylitis (AS) and some forms of psoriatic arthritis (PsA), is characterized by shared pathogenic, clinical, and radiographic features, such as axial arthritis, with or without peripheral arthritis, enthesitis, dactylitis, and an association with HLA-B27 (2). PsA is strongly associated with psoriasis (PsO), a chronic inflammatory skin disease, with approximately 6% to 42% of patients with PsO developing PsA over time (3). Uveitis is among the most serious extra-articular manifestations in axSpA due to its potential for irreversible vision loss, accounting for up to 10% to 15% of legal blindness in developed countries (4, 5). Uveitis affects approximately 25%–40% of patients with AS, up to 10% of those with PsA, and 2%–5% of individuals with PsO (6). When inadequately treated or recurrent, uveitis may lead to complications including macular edema, cataracts, retinal detachment, and, ultimately, permanent visual impairment (4).

The axSpA involves a complex interplay of cytokines, including tumor necrosis factor-α (TNF-α), interleukin (IL)-6, IL-12, IL-23, and IL-17A, which mediate both musculoskeletal and extra-articular inflammation (7). TNF-α contributes to intraocular inflammation by inducing blood–retinal barrier disruption through activation of the NF-κB and MAPK signaling pathways (8). In contrast, IL-6, IL-12, and IL-23 activate the Janus kinase (JAK)–signal transducer and activator of transcription (STAT) pathway, promoting the differentiation of Th1 and Th17 cells (9). These mechanistic insights have led to the development of targeted therapies, with tumour necrosis factor inhibitors (TNFi) and JAK inhibitors (JAKi) both demonstrating efficacy in axSpA. Current guidelines by EULAR and GRAPPA recommend their use as second-line therapies in patients with inadequate response to conventional treatment (10, 11).

Despite their inclusion in treatment guidelines, comparative data on the relative effectiveness of TNFi and JAKi in reducing the risk of uveitis remain limited. Previous studies have established the efficacy of monoclonal antibody TNFi in preventing uveitis onset and reducing recurrence in patients with AS (12–15). In contrast, current evidence on JAKi largely stems from underpowered investigations, including case reports, small observational studies, and the prematurely terminated HUMBOLDT trial, all of which lack robust control groups (16–19). Recent safety concerns surrounding JAKi have further complicated therapeutic decision-making in clinical practice (20, 21). Moreover, it remains unclear whether the distinct immunologic mechanisms of TNFi and JAKi may differentially influence the risk of uveitis. These knowledge gaps underscore the urgent need for large-scale, head-to-head comparative studies evaluating the impact of these targeted therapies on uveitis risk.

To address this knowledge gap, we conducted a large-scale, real-world comparative study to evaluate the risk of incident uveitis among patients with psoriatic disease and AS treated with either TNFi or JAKi.

## 2 Methods

This retrospective cohort study utilized data from the TriNetX Network (https://trinetx.com/), a global health research platform that provides access to de-identified electronic health records (EHRs), encompassing diagnoses, procedures, medications, laboratory results, and genomic data. Data were obtained from a subset of the TriNetX database, comprising 68 healthcare organizations (HCOs) and approximately 116 million patients across the United States. All data analyses were conducted in November 2024. This study was approved by the Institutional Review Board (IRB) of Chung Shan Medical University Hospital (Approval No. CS1-24069) and was conducted in accordance with the principles of the Declaration of Helsinki.

### 2.1 Participants

We included adult patients (≥18 years) who had at least two outpatient diagnoses of AS, PsO, or PsA between January 1, 2000, and December 31, 2023. Diagnoses were identified using the International Classification of Diseases, 10th Revision, Clinical Modification (ICD-10-CM) codes, which include AS (M45, M46), PsO (L40.0, L40.1, L40.2, L40.3, L40.4, L40.8, L40.9), and PsA (L40.5). These diagnostic codes have been previously validated in studies utilizing administrative claims data and electronic health records (22–24).

### 2.2 Exposure

In this target trial emulation study, exposure was defined as the first prescription of either a JAKi or TNFi during the study period. The date of the first prescription was designated as the index date. To reduce prevalent user bias, patients with any prior prescription record of JAKi or TNFi before January 1, 2016, were excluded. The JAKi cohort included patients who received a new prescription for any JAKi, identified using the Anatomical Therapeutic Chemical (ATC) classification code L04AA, encompassing tofacitinib, baricitinib, and upadacitinib. The TNFi cohort comprised patients who initiated therapy with any TNFi, specifically TNF monoclonal antibodies (TNF mAbs), identified by ATC code L04AB, including infliximab, adalimumab, certolizumab pegol, and golimumab (25). We further excluded patients who switched between or concurrently used both JAKi and TNFi at any time after January 1, 2016. Additionally, patients with a history of uveitis prior to the index date were also excluded.

### 2.3 Outcome

The primary outcome of interest was the occurrence of incident uveitis. The diagnostic subtypes of uveitis include chorioretinal inflammation (ICD-10-CM H30), iridocyclitis (ICD-10-CM H20), pan-uveitis (ICD-10-CM H44.11), sympathetic uveitis (ICD-10-CM H44.13), ophthalmia nodosa (ICD-10-CM H16.24), and retinal vasculitis (ICD-10-CM H35.06). These ICD-10-CM codes are widely recognized and commonly used in tertiary care settings to ensure accurate diagnosis (26, 27). To minimize potential misclassification of preexisting uveitis and to ensure that identified cases represented incident events, a washout period of 30 days after the index date was implemented. Participants were subsequently followed from the end of this washout period until the occurrence of uveitis or the end of the study period, whichever came first.

### 2.4 Covariate

Baseline characteristics of eligible patients were extracted from the TriNetX database. Key demographic variables included age, sex, ethnicity, race, body mass index (BMI), socioeconomic status, and lifestyle factors. Pre-existing comorbidities identified in previous studies, as well as prior use of nonsteroidal anti-inflammatory drugs (NSAIDs) and conventional synthetic disease-modifying antirheumatic drugs (csDMARDs), were also recorded (28, 29). Laboratory markers, including C-reactive protein (CRP) and erythrocyte sedimentation rate (ESR), were considered potential confounders (30). Definitions of all variables are detailed in **Supplementary Table 7**. Exposure variables were defined using data from the 12-month period preceding the index date. To further evaluate the potential impact of unmeasured confounding, we calculated E-values for the association between JAKi use and incident uveitis.

### 2.5 Statistical analysis

To minimize confounding, we used the built-in 1:1 propensity score matching (PSM) function in TriNetX. A standardized mean difference (SMD) of less than 0.1 was considered indicative of adequate covariate balance between matched groups (31). After confirming the proportional hazards assumption, we applied Cox proportional hazards models to estimate hazard ratio (HR) and 95% confidence interval (CI) for the risk of uveitis. The Kaplan–Meier method was used to estimate the cumulative incidence of uveitis, and differences between groups were assessed using the log-rank test. Statistical significance was defined as a two-sided p-value of <0.05.

We conducted the following prespecified subgroup analyses. Stratification by age, sex, and race was performed to assess potential effect modification. Additional subgroup analyses were based on underlying disease type, including AS and psoriatic diseases. To further explore treatment heterogeneity, patients were stratified according to the specific type of JAKi received. The impact of concomitant use of csDMARDs during the treatment period was assessed to determine whether it modified the effect of JAKi or TNFi therapy on the risk of uveitis. Lastly, analyses stratified by ESR and CRP levels were conducted to assess whether baseline inflammatory severity influenced treatment outcomes.

### 2.6 Sensitivity analyses

We also conducted several sensitivity analyses to evaluate the robustness of our results. To align with the per-protocol approach commonly used in clinical trials, we restricted the analysis to patients who had received at least two prescriptions of the study drug to ensure treatment adherence. Additionally, to account for treatment switching, we refined the exclusion criteria by excluding patients who received both treatments concurrently within the first three months of follow-up. Given that adalimumab is the only FDA-approved biologic for uveitis, we conducted a sensitivity analysis using it as the reference group (32). Lastly, we performed additional analyses using data from the TriNetX Global Collaborative Network to further assess the consistency of our results. All analyses were conducted within the TriNetX platform.

## 3 Results

**Figure 1:**
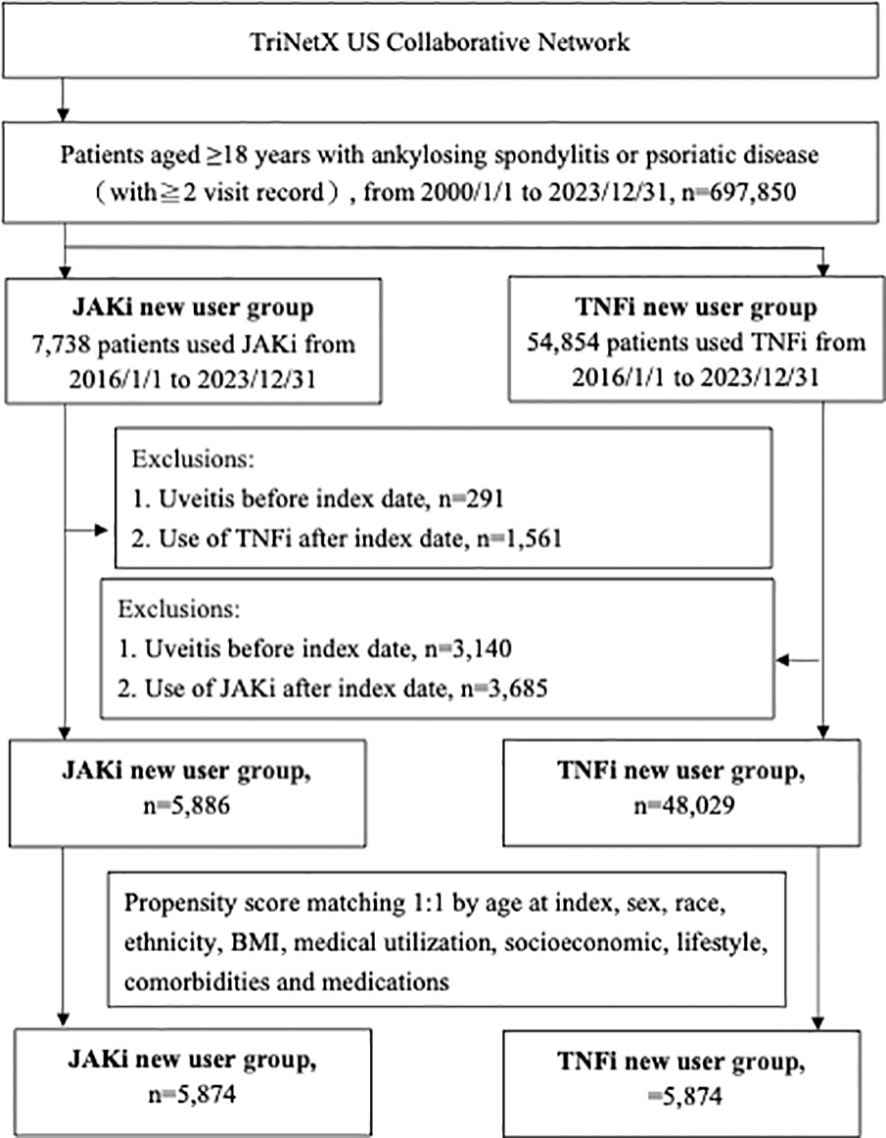
Participant selection flowchart of study cohort selection.

From January 1, 2000, to December 31, 2023, a total of 697,850 adult patients with AS, PsO, or PsA were identified from the TriNetX US Collaborative Network. Between 2016 and 2023, 62,592 patients without prior exposure to JAKi or TNFi were deemed eligible. After applying the exclusion criteria, 53,915 patients who initiated treatment with JAKi or TNFi at one of the 68 participating HCOs were included and subsequently categorized into JAKi and TNFi cohorts (**Figure 1**). After PSM matching, the baseline characteristics of 5,874 patients in each group are presented in **Supplementary Table 1**, showing well-balanced covariates across all measures (SMD < 0.1). The mean (SD) age of JAKi participants was 53.5 (14.3) years, with a mean BMI of 31.6 (7.9). Among the patients for whom sex data was available, 3,893 (66.3%) were women.

**Figure 2:**
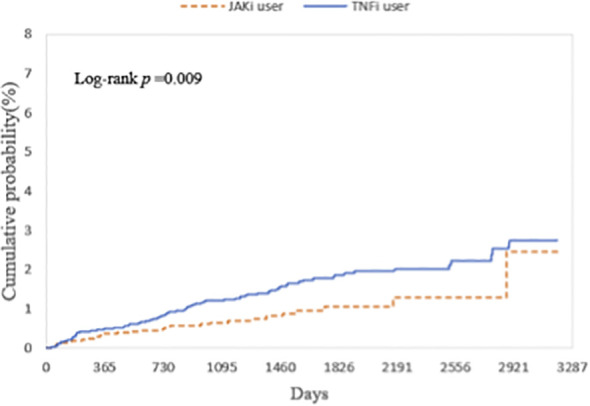
Kaplan-Meier curves showing the cumulative probability (%) of uveitis, comparing the risk of incident uveitis events in patients with autoimmune diseases treated with JAKi compared to TNFi over a 9-year follow-up period. JAKi, Janus kinase Inhibitors; TNFi, tumor necrosis factor-alpha inhibitors.

**Table 1** presents the HR for uveitis from four multivariable Cox proportional hazards models. In all models, patients receiving JAKi therapy had a consistently lower risk of uveitis compared with those receiving TNFi therapy. The HR were 0.630 (95% CI, 0.418–0.948) in Model 1, The HR were 0.530 (95% CI, 0.356–0.789) in Model 2, The HR were 0.649 (95% CI, 0.43–0.979) in Model 3, and The HR were 0.596 (95% CI, 0.403–0.880) in Model 4. The E-values for the point estimates ranged from 2.454 to 3.180, and those for the lower bounds of the confidence intervals ranged from 1.169 to 1.850. These values indicate that an unmeasured confounder would need to be associated with both the exposure and the outcome by a risk ratio of at least 2.5 to fully explain away the observed associations, beyond the measured covariates already included in our models. **Figure 2** illustrates that the cumulative incidence of uveitis over a nine-year follow-up period, following a washout period after the index date, was significantly lower in patients receiving JAKi therapy compared with those receiving TNFi therapy (log-rank test, *P* = 0.009).

**Table 1:** Risk of incident uveitis events in patients with autoimmune disease treated with JAKi compared to TNFi over a 9-year follow-up period.

Group	Patients in cohort	Patients with outcome	Model 1 hazard ratio* (95% CI)	Model 2 hazard ratio (95% CI)	Model 3 hazard ratio (95% CI)	Model 4 hazard ratio (95% CI)
JAKi user	5,874	37	0.63 (0.418,0.948)	0.53 (0.356,0.789)	0.649(0.43,0.979)	0.596(0.403, 0.880)
TNFi user	5,874	86	reference	reference	reference	reference
E-value (CI)			2.553(1.295)	3.180(1.850)	2.454(1.169)	2.744(1.530)

*Hazard ratio for outcomes among JAKi group compared to TNFi group subjects (after propensity score matching).

95% CI, 95% confidence interval.

Among patients aged ≥51 years (HR, 0.43; 95% CI, 0.24–0.79) and in White individuals (HR, 0.59; 95% CI, 0.38–0.93), JAKi therapy was associated with a significantly lower risk of uveitis compared with TNFi therapy (**Supplementary Figure 1**). No significant difference in uveitis risk was observed between patients with AS and those with psoriatic disease (**Supplementary Figure 2**), or across different JAKi subtypes (**Supplementary Figure 3**). In stratified analyses based on csDMARD use during follow-up, JAKi therapy remained protective in both subgroups. The HR was 0.50 (95% CI, 0.28–0.92) among patients receiving csDMARDs and 0.56 (95% CI, 0.33–0.94) among those not receiving csDMARDs (**Supplementary Figure 4**). Inflammatory burden also appeared to modify treatment effects. JAKi therapy was associated with a significantly lower risk of uveitis in patients with elevated CRP (≥3 mg/L; HR, 0.50; 95% CI, 0.26–0.96) and elevated ESR (≥20 mm/h; HR, 0.41; 95% CI, 0.19–0.87). In contrast, no significant difference was observed in patients with lower inflammatory levels (**Supplementary Figure 5**).

In the on-treatment analysis, which was restricted to patients with ≥2 prescriptions, JAKi therapy remained associated with a significantly lower risk of uveitis compared with TNFi (HR, 0.605; 95% CI, 0.385–0.952; **Supplementary Table 3**). After excluding patients who received both therapies within the first three months, the results remained consistent (HR, 0.675; 95% CI, 0.471–0.969; **Supplementary Table 4**). Using adalimumab as the reference group yielded similar findings (**Supplementary Table 5**). The results were also consistent in analyses utilizing data from the TriNetX Global Collaborative Network (**Supplementary Table 6**).

## 4 Discussion

In this large-scale cohort study of patients with AS, PsO, or PsA, initiation of JAKi therapy was associated with a lower risk of incident uveitis compared with TNFi therapy. These findings were consistent across all four sensitivity analyses, supporting the robustness of the primary results. Subgroup analyses showed that the protective effect of JAKi therapy against uveitis was more pronounced in older adults, White individuals, and patients with elevated inflammatory markers (CRP ≥3 mg/L or ESR ≥20 mm/h). Notably, this association remained regardless of concomitant csDMARD use.

To our knowledge, this is the first study to investigate the association between targeted synthetic disease-modifying antirheumatic drugs (tsDMARDs) and biologic agents and the risk of uveitis using large-scale real-world data. It builds on prior research into the ocular effects of JAKi, further supporting their potential role in inflammatory eye disease management (16–19). Miserocchi et al. reported reduced intraocular inflammation and relapse rates in refractory juvenile idiopathic arthritis (JIA)-associated uveitis treated with JAKi (16). Similarly, Wen et al. observed clinical improvement in patients with refractory non-infectious uveitis or scleritis following JAKi therapy (17). In a prospective cohort study conducted within the international AutoInflammatory Disease Alliance (AIDA) Network, ocular flare incidence dropped from 125 to 28.6 events per 1,000 person-months after JAKi initiation (IRR 4.37, 95% CI 1.3–14.7; p=0.02) (18). Additionally, the phase 2 HUMBOLDT trial showed filgotinib significantly reduced treatment failure in active non-infectious uveitis versus placebo (37.5% vs. 67.6%; p=0.006) (19). By contrast, a network meta-analysis of axial spondyloarthritis by Bechman et al. found that TNF monoclonal antibodies, JAKi, and IL-17 inhibitors all reduced the risk of anterior uveitis, with no significant differences between them (33). However, SUCRA ranking suggested the lowest risk with TNF monoclonal antibodies, followed by JAK inhibitors, IL-17 inhibitors, and finally etanercept (33). These discrepancies highlight the need for future head-to-head comparative and randomized studies to more clearly delineate the relative effectiveness of JAKi compared to established biologic therapies in the prevention and management of uveitis across diverse patient populations.

Neverteless, potential channelling bias should be considered when interpreting our findings. In clinical practice, TNFi are typically used as first-line biologics for AS and PsA, particularly in patients with axial involvement or a prior history of uveitis (10). In contrast, JAKi are more commonly reserved for patients with more severe disease or those who have not responded adequately to conventional therapies (34). To minimize potential bias, we restricted the cohort to patients without a prior diagnosis of uveitis and adjusted for disease severity proxies, including baseline CRP, ESR, and prior exposure to conventional synthetic DMARDs within the year before treatment initiation.

The lower risk of uveitis observed among patients receiving JAK inhibitors suggests potential mechanistic differences compared with TNF inhibitors. JAK inhibitors modulate multiple inflammatory pathways by suppressing key cytokines, including IL-6, IL-12, and IL-23, which are implicated in the pathogenesis of both spondyloarthritis and ocular inflammation (9, 35). Given the pivotal role of IL-23 in Th17-mediated immune responses, the broader cytokine inhibition achieved by JAK inhibitors may contribute to reduced uveitis risk (36, 37). Furthermore, JAK inhibitors, being small molecules, may penetrate the blood-retina barrier more effectively than biologics, allowing for direct modulation of intraocular inflammation (38). However, these remain theoretical hypotheses that require validation through future research.

This study has several important implications. When randomized controlled trials are prohibitively expensive or time-consuming, emulated target trials using observational data provide a valuable source of real-world evidence on the effectiveness of interventions (39). Further studies employing similar methodologies are warranted to validate and build upon these findings. Additionally, we were unable to assess detailed clinical data, such as uncoded measures of disease activity, joint pain, and medication dosages. Incorporating more detailed clinical variables in future observational studies will help mitigate potential residual confounding and enhance the validity of comparative effectiveness research. While our study focused on the long-term risk of uveitis, emerging concerns have been raised regarding the safety of prolonged JAKi use, particularly in older adults (20). The FDA has issued black box warnings highlighting potential risks, including major adverse cardiovascular events and malignancies (20, 21). Further studies are warranted to evaluate the long-term safety profile of JAKi and to inform clinical decision-making.

The strengths of our study include the use of a large real-world database, a new-user design, an active comparator approach, multiple sensitivity analyses supporting the main findings, and stratified analyses to mitigate potential confounding. However, several limitations should be acknowledged. First, reliance on electronic health records may introduce coding errors or misclassification, which could potentially affect the accuracy of the study findings. Second, the TriNetX database lacks detailed information on medication dosage, disease severity, smoking status, and imaging findings, which may limit the precision of linking drug exposure to clinical outcomes. Moreover, due to the absence of imaging data, we were unable to distinguish between radiographic and non-radiographic axSpA. Third, a further limitation is the inability to restrict diagnoses to those made exclusively by ophthalmologists, as the TriNetX platform does not capture physician specialty information. Fourth, the majority of patients in our cohort were White. Although race and ethnicity were balanced between treatment groups after propensity score matching (SMD <0.1), the limited racial and ethnic diversity may restrict the external validity and generalizability of our results. Finally, although the TriNetX database is extensive, it only includes outpatient data from HCOs that are part of the TriNetX network. Patients who discontinue treatment or seek care outside the network are lost to follow-up, which limits the generalizability of our findings, particularly in regions with less comprehensive healthcare coverage.

## 5 Conclusions

In this real-world cohort study, JAK inhibitor therapy was associated with a reduced risk of incident uveitis compared with TNF inhibitors among patients with ankylosing spondylitis, psoriasis, or psoriatic arthritis. These findings support a potential therapeutic role for JAK inhibitors in mitigating ocular inflammatory risk in this population. Further prospective studies are warranted to validate these results and elucidate the underlying mechanisms.

## Data Availability

The original contributions presented in the study are included in the article/**Supplementary Material**. Further inquiries can be directed to the corresponding authors.
