# Supplementary figures and images for: Comparative risk of uveitis with Janus kinase inhibitors versus tumor necrosis factor inhibitors in ankylosing spondylitis and psoriatic diseases: a target trial emulation study
Source: Front Immunol. 2025 Oct 24;16:1673970. doi: 10.3389/fimmu.2025.1673970 (PMC12592076; doi:10.3389/fimmu.2025.1673970)

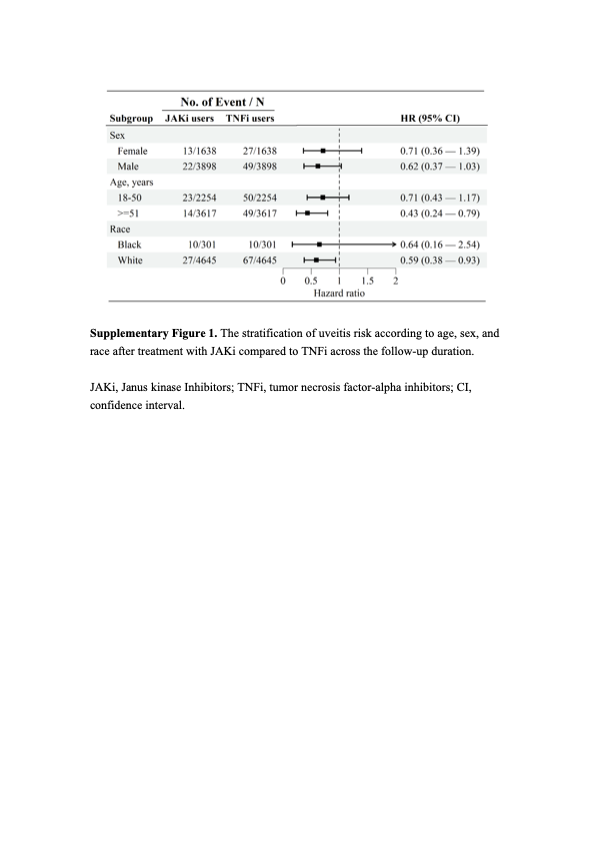

Supplement: Supplementary file 1 [file Image1.tiff]

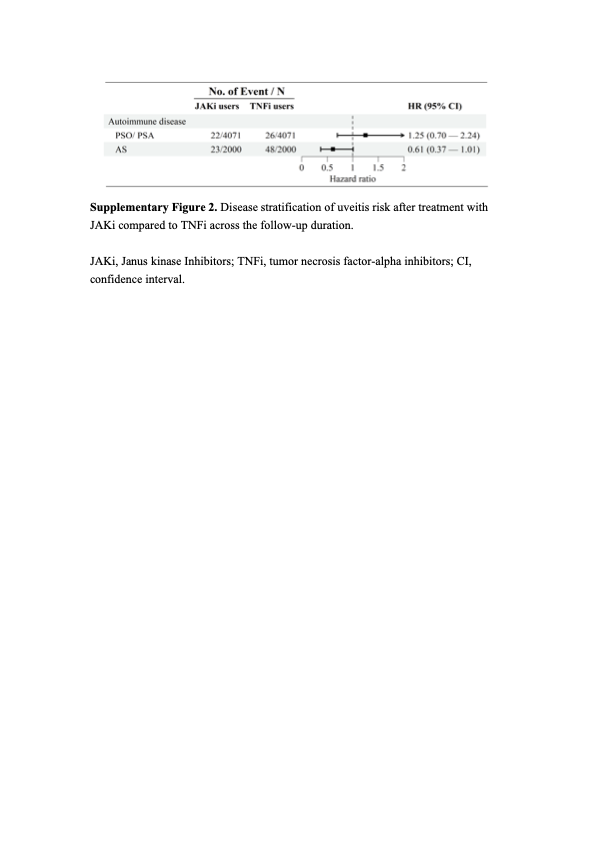

Supplement: Supplementary file 2 [file Image2.tiff]

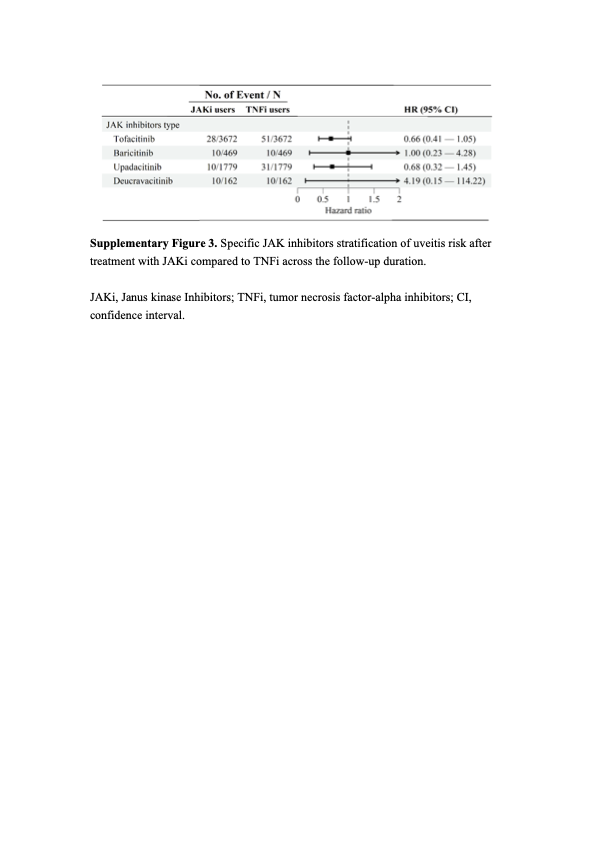

Supplement: Supplementary file 3 [file Image3.tiff]

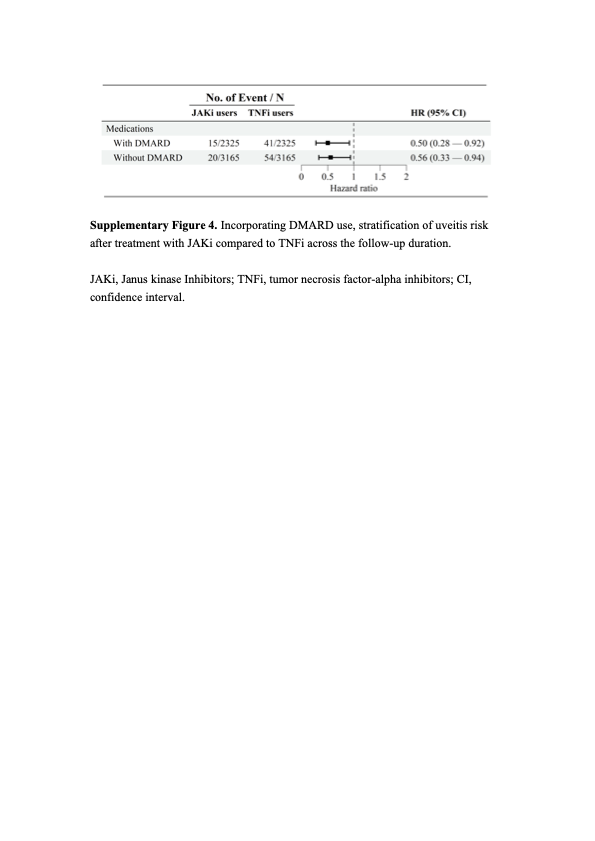

Supplement: Supplementary file 4 [file Image4.tiff]

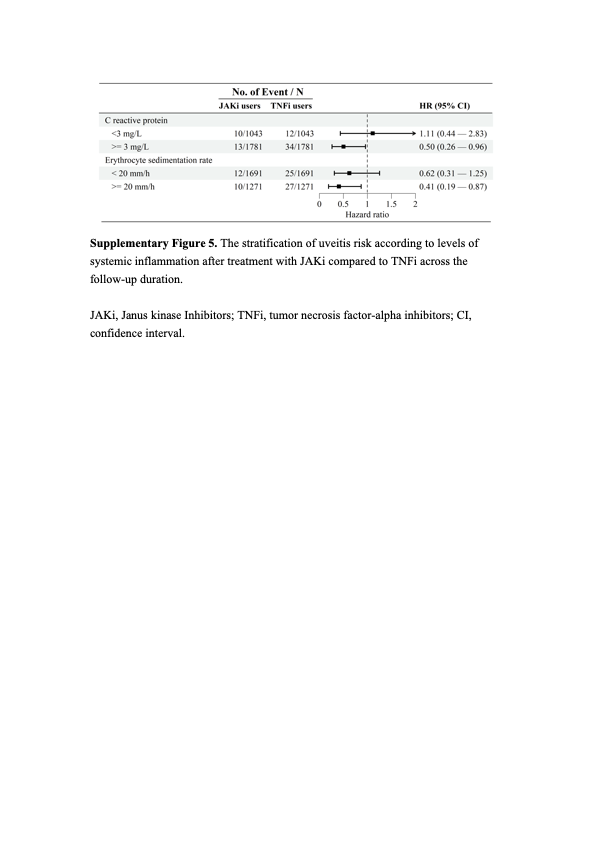

Supplement: Supplementary file 5 [file Image5.tiff]
