# Supplementary material for: Comparative risk of uveitis with Janus kinase inhibitors versus tumor necrosis factor inhibitors in ankylosing spondylitis and psoriatic diseases: a target trial emulation study
Source: Front Immunol. 2025 Oct 24;16:1673970. doi: 10.3389/fimmu.2025.1673970 (PMC12592076; doi:10.3389/fimmu.2025.1673970)
Supplement: Supplementary file 6 [file Table1.docx]

**Supplementary Table 1.**

Baseline Characteristics of Autoimmune Disease Patients with Propensity Score-Matched Comparison of JAKi and TNFi.

|  | Before PSM | | | After PSM | | |
| --- | --- | --- | --- | --- | --- | --- |
|  | JAKi user | TNFi user | SMD | JAKi user | TNFi user | SMD |
| **N** | 5,886 | 48,029 |  | 5,874 | 5,874 |  |
| Age at index (Mean±SD) | 53.5±14.3 | 48.2±15.3 | 0.3625 | 53.5±14.3 | 54.0±14.5 | 0.0330 |
| **Sex** |  |  |  |  |  |  |
| Female | 3904(66.3%) | 25586(53.3%) | 0.2687 | 3893(66.3%) | 3975(67.7%) | 0.0297 |
| Male | 1722(29.3%) | 20921(43.6%) | 0.3006 | 1722(29.3%) | 1627(27.7%) | 0.0358 |
| **Ethnicity** |  |  |  |  |  |  |
| Hispanic or Latino | 374(6.4%) | 3245(6.8%) | 0.0163 | 373(6.4%) | 382(6.5%) | 0.0062 |
| Not Hispanic or Latino | 4283(72.8%) | 34801(72.5%) | 0.0069 | 4274(72.8%) | 4267(72.6%) | 0.0027 |
| Unknown Ethnicity | 1229(20.9%) | 9983(20.8%) | 0.0023 | 1227(20.9%) | 1225(20.9%) | 0.0008 |
| **Race** |  |  |  |  |  |  |
| American Indian or Alaska Native | 28(0.5%) | 314(0.7%) | 0.0238 | 28(0.5%) | 26(0.4%) | 0.0050 |
| Asian | 153(2.6%) | 1222(2.5%) | 0.0035 | 152(2.6%) | 147(2.5%) | 0.0054 |
| Black or African American | 310(5.3%) | 2809(5.8%) | 0.0254 | 310(5.3%) | 307(5.2%) | 0.0023 |
| Native Hawaiian or Other Pacific Islander | 12(0.2%) | 95(0.2%) | 0.0014 | 12(0.2%) | 13(0.2%) | 0.0037 |
| White | 4658(79.1%) | 37184(77.4%) | 0.0416 | 4648(79.1%) | 4632(78.9%) | 0.0067 |
| Other Race | 166(2.8%) | 1721(3.6%) | 0.0434 | 166(2.8%) | 163(2.8%) | 0.0031 |
| Unknown Race | 559(9.5%) | 4684(9.8%) | 0.0087 | 558(9.5%) | 586(10.0%) | 0.0161 |
| **BMI** | 31.5±7.9 | 31.4±7.9 | 0.0226 | 31.6±7.9 | 31.9±8.0 | 0.0406 |
| At most 18.5 kg/m2 | 103(1.8%) | 678(1.4%) | 0.0271 | 102(1.7%) | 95(1.6%) | 0.0093 |
| 18.5-24 kg/m2 | 707(12.0%) | 5026(10.5%) | 0.0490 | 704(12.0%) | 715(12.2%) | 0.0057 |
| 24-30 kg/m2 | 1562(26.5%) | 10470(21.8%) | 0.1108 | 1554(26.5%) | 1593(27.1%) | 0.0150 |
| At least 30 kg/m2 | 2298(39.0%) | 14669(30.5%) | 0.1792 | 2292(39.0%) | 2367(40.3%) | 0.0261 |
| **Medical utilization** |  |  |  |  |  |  |
| Office or Other Outpatient Services | 3614(61.4%) | 26646(55.5%) | 0.1204 | 3605(61.4%) | 3633(61.8%) | 0.0098 |
| Hospital Inpatient Services | 556(9.4%) | 2182(4.5%) | 0.1931 | 551(9.4%) | 498(8.5%) | 0.0316 |
| Emergency Department Services | 932(15.8%) | 5013(10.4%) | 0.1603 | 925(15.7%) | 891(15.2%) | 0.0160 |
| **Socioeconomic and psychosocial circumstances** | 76(1.3%) | 482(1.0%) | 0.0270 | 76(1.3%) | 71(1.2%) | 0.0077 |
| **Lifestyle** |  |  |  |  |  |  |
| Tobacco use | 95(1.6%) | 838(1.7%) | 0.0102 | 95(1.6%) | 80(1.4%) | 0.0211 |
| Nicotine dependence | 409(6.9%) | 2949(6.1%) | 0.0327 | 409(7.0%) | 406(6.9%) | 0.0020 |
| Alcohol related disorders | 75(1.3%) | 777(1.6%) | 0.0288 | 75(1.3%) | 68(1.2%) | 0.0109 |
| **Comorbidities** |  |  |  |  |  |  |
| Hypertensive diseases | 1912(32.5%) | 10732(22.3%) | 0.2288 | 1904(32.4%) | 1925(32.8%) | 0.0076 |
| Diabetes mellitus | 869(14.8%) | 5038(10.5%) | 0.1290 | 866(14.7%) | 883(15.0%) | 0.0081 |
| Hyperlipidemia | 1476(25.1%) | 8597(17.9%) | 0.1754 | 1470(25.0%) | 1522(25.9%) | 0.0203 |
| Neoplasms | 909(15.4%) | 5670(11.8%) | 0.1062 | 906(15.4%) | 912(15.5%) | 0.0028 |
| Chronic kidney disease (CKD) | 333(5.7%) | 1360(2.8%) | 0.1405 | 332(5.7%) | 301(5.1%) | 0.0234 |
| Other rheumatoid arthritis | 1550(26.3%) | 5086(10.6%) | 0.4144 | 1538(26.2%) | 1545(26.3%) | 0.0027 |
| Rheumatoid arthritis | 641(10.9%) | 1209(2.5%) | 0.3396 | 632(10.8%) | 552(9.4%) | 0.0453 |
| Systemic lupus erythematosus (SLE) | 148(2.5%) | 367(0.8%) | 0.1382 | 148(2.5%) | 128(2.2%) | 0.0225 |
| Sjögren syndrome | 207(3.5%) | 571(1.2%) | 0.1540 | 204(3.5%) | 194(3.3%) | 0.0094 |
| Behçet's disease | 13(0.2%) | 67(0.1%) | 0.0192 | 13(0.2%) | 13(0.2%) | 0.0000 |
| Inflammatory bowel disease, | 568(9.7%) | 4509(9.4%) | 0.0089 | 567(9.7%) | 553(9.4%) | 0.0081 |
| Systemic sclerosis | 17(0.3%) | 64(0.1%) | 0.0339 | 17(0.3%) | 15(0.3%) | 0.0065 |
| Multiple Sclerosis (MS) | 38(0.6%) | 55(0.1%) | 0.0864 | 36(0.6%) | 34(0.6%) | 0.0044 |
| Diseases of liver | 388(6.6%) | 2342(4.9%) | 0.0738 | 387(6.6%) | 403(6.9%) | 0.0109 |
| Heart failure | 269(4.6%) | 707(1.5%) | 0.1817 | 266(4.5%) | 223(3.8%) | 0.0367 |
| Ischemic heart diseases | 460(7.8%) | 2115(4.4%) | 0.1428 | 457(7.8%) | 456(7.8%) | 0.0006 |
| Cerebrovascular diseases | 169(2.9%) | 803(1.7%) | 0.0806 | 169(2.9%) | 170(2.9%) | 0.0010 |
| Chronic obstructive pulmonary disease(COPD) | 328(5.6%) | 1206(2.5%) | 0.1559 | 326(5.6%) | 322(5.5%) | 0.0030 |
| Herpes zoster | 66(1.1%) | 306(0.6%) | 0.0519 | 65(1.1%) | 65(1.1%) | 0.0000 |
| **Medications** |  |  |  |  |  |  |
| NSAIDs | 2404(40.8%) | 15325(31.9%) | 0.1865 | 2397(40.8%) | 2511(42.7%) | 0.0394 |
| Hydroxychloroquine | 517(8.8%) | 1572(3.3%) | 0.2331 | 511(8.7%) | 508(8.6%) | 0.0018 |
| Methotrexate | 1303(22.1%) | 8348(17.4%) | 0.1197 | 1301(22.1%) | 1383(23.5%) | 0.0333 |
| Sulfasalazine | 393(6.7%) | 2205(4.6%) | 0.0906 | 391(6.7%) | 395(6.7%) | 0.0027 |
| Leflunomide | 497(8.4%) | 1238(2.6%) | 0.2592 | 488(8.3%) | 473(8.1%) | 0.0093 |
| Azathioprine | 109(1.9%) | 531(1.1%) | 0.0619 | 107(1.8%) | 101(1.7%) | 0.0077 |
| **Laboratory tests** |  |  |  |  |  |  |
| C reactive protein |  |  |  |  |  |  |
| At most 1 mg/L | 602(10.2%) | 3404(7.1%) | 0.1118 | 601(10.2%) | 598(10.2%) | 0.0017 |
| 1-3 mg/L | 613(10.4%) | 3616(7.5%) | 0.1011 | 610(10.4%) | 639(10.9%) | 0.0160 |
| At least 3 mg/L | 1695(28.8%) | 9425(19.6%) | 0.2154 | 1688(28.7%) | 1747(29.7%) | 0.0221 |
| Erythrocyte sedimentation rate |  |  |  |  |  |  |
| At most 20 mm/h | 1646(28.0%) | 10404(21.7%) | 0.1463 | 1643(28.0%) | 1728(29.4%) | 0.0320 |
| At least 20 mm/h | 1191(20.2%) | 6332(13.2%) | 0.1898 | 1186(20.2%) | 1174(20.0%) | 0.0051 |

*PSM (matching include Age at index, Sex, Ethnicity, Race, BMI, Medical utilization, Socioeconomic, Lifestyle, Comorbidities and Medications)

CKD: Chronic kidney disease; COPD: Chronic obstructive pulmonary disease; SMD: standardized mean difference.

**Table 2.**

Risk of incident uveitis events in patients with autoimmune disease treated with JAKi compared to TNFi over a 9-year follow-up period.

|  | Patients in cohort | Patients with outcome | Model 1  Hazard ratio*  （95% CI） | Model 2  Hazard ratio  （95% CI） | Model 3  Hazard ratio  （95% CI） | Model 4  Hazard ratio  （95% CI） |
| --- | --- | --- | --- | --- | --- | --- |
| JAKi user | 5,874 | 37 | 0.63 （0.418,0.948） | 0.53 （0.356,0.789） | 0.649（0.43,0.979） | 0.596（0.403, 0.880） |
| TNFi user | 5,874 | 86 | reference | reference | reference | reference |
| E-value (CI) |  |  | 2.553(1.295) | 3.180(1.850) | 2.454(1.169) | 2.744(1.530) |

*Hazard ratio for outcomes among JAKi group compared to TNFi group subjects （after propensity score matching）.

95% CI, 95% confidence interval.

Model 1: propensity score matching include Age at index, Sex, Ethnicity, Race, BMI, Medical utilization, Socioeconomic and Lifestyle.

Model 2: propensity score matching include Age at index, Sex, Ethnicity, Race, BMI, Medical utilization, Socioeconomic, Lifestyle and Comorbidities.

Model 3 propensity score matching include Age at index, Sex, Ethnicity, Race, BMI, Medical utilization, Socioeconomic, Lifestyle, Comorbidities and Medications.

Model 4 propensity score matching include Age at index, Sex, Ethnicity, Race, BMI, Medical utilization, Socioeconomic, Lifestyle, Comorbidities, Medications and Laboratory.

**Supplementary Table 3.**

The on-treatment design for the risk of uveitis events among patients with autoimmune diseases undergoing treatment with JAKi compared to TNFi.

|  | Patients in cohort | Patients with outcome | Hazard ratio*  （95% CI） |
| --- | --- | --- | --- |
| JAKi user | 3,866 | 28 | 0.605（0.385, 0.952） |
| TNFi user | 3,866 | 60 | reference |

*Hazard ratio for outcomes among JAKi group compared to TNFi group subjects （after propensity score matching）.

95% CI, 95% confidence interval.

propensity score matching include Age at index, Sex, Ethnicity, Race, BMI, Medical utilization, Socioeconomic, Lifestyle, Comorbidities, Medications and Laboratory.

JAKi, Janus kinase Inhibitors; TNFi, tumor necrosis factor-alpha inhibitors; CI, confidence interval.

**Supplementary Table 4.**

The drug-switch for the risk of uveitis events among patients with autoimmune diseases undergoing treatment with JAKi compared to TNFi.

|  | Patients in cohort | Patients with outcome | Hazard ratio*  （95% CI） |
| --- | --- | --- | --- |
| JAKi user | 6,426 | 50 | 0.675（0.471, 0.969） |
| TNFi user | 6,426 | 72 | reference |

*Hazard ratio for outcomes among JAKi group compared to TNFi group subjects （after propensity score matching）.

95% CI, 95% confidence interval.

propensity score matching include Age at index, Sex, Ethnicity, Race, BMI, Medical utilization, Socioeconomic, Lifestyle, Comorbidities, Medications and Laboratory.

JAKi, Janus kinase Inhibitors; TNFi, tumor necrosis factor-alpha inhibitors; CI, confidence interval.

**Supplementary Table 5.**

Results of the risk of uveitis events among patients with autoimmune diseases undergoing treatment with JAKi compared to Adalimumab.

|  | Patients in cohort | Patients with outcome | Hazard ratio*  （95% CI） |
| --- | --- | --- | --- |
| JAKi user | 5,877 | 39 | 0.621 （0.424, 0.911） |
| Adalimumab user | 5,877 | 87 | reference |

*Hazard ratio for outcomes among JAKi group compared to TNFi group subjects （after propensity score matching）.

95% CI, 95% confidence interval.

propensity score matching include Age at index, Sex, Ethnicity, Race, BMI, Medical utilization, Socioeconomic, Lifestyle, Comorbidities, Medications and Laboratory.

JAKi, Janus kinase Inhibitors; CI, confidence interval.

**Supplementary Table 6.**

The Global Collaborative Network analysis on the risk of uveitis events among patients with autoimmune diseases treated with JAKi compared to TNFi.

|  | Patients in cohort | Patients with outcome | Hazard ratio*  （95% CI） |
| --- | --- | --- | --- |
| JAKi user | 6,226 | 40 | 0.668（0.456, 0.979） |
| TNFi user | 6,226 | 84 | reference |

*Hazard ratio for outcomes among JAKi group compared to TNFi group subjects （after propensity score matching）.

95% CI, 95% confidence interval.

propensity score matching include Age at index, Sex, Ethnicity, Race, BMI, Medical utilization, Socioeconomic, Lifestyle, Comorbidities, Medications and Laboratory.

JAKi, Janus kinase Inhibitors; TNFi, tumor necrosis factor-alpha inhibitors; CI, confidence interval.

**Supplementary Table 7.**

Definitions of covariates.

| **Co-variates (within 1 years before index date)** | **Code(s)** |
| --- | --- |
| **Sex** |  |
| Female |  |
| Male |  |
| **Ethnicity** |  |
| Hispanic or Latino |  |
| Not Hispanic or Latino |  |
| Unknown Ethnicity |  |
| **Race** |  |
| American Indian or Alaska Native |  |
| Asian |  |
| Black or African American |  |
| Native Hawaiian or Other Pacific Islander |  |
| White |  |
| Other Race |  |
| **BMI** |  |
| **Medical utilization** |  |
| Office or Other Outpatient Services | 1013626 |
| Hospital Inpatient Services | 1013659 |
| Emergency Department Services | 1013711 |
| **Socioeconomic and psychosocial circumstances** | Z55-Z65 |
| Problems related to education and literacy | Z55 |
| Problems related to employment and unemployment | Z56 |
| Occupational exposure to risk factors | Z57 |
| Problems related to housing and economic circumstances | Z59 |
| **Lifestyle** |  |
| Tobacco use | Z72.0 |
| Nicotine dependence | F17 |
| Alcohol related disorders | F10 |
| **Comorbidities** |  |
| Hypertensive diseases | I10-I16 |
| Diabetes mellitus | E08-E13 |
| Hyperlipidemia | E78 |
| Neoplasms | C00-D49 |
| Chronic kidney disease (CKD) | N18 |
| Other rheumatoid arthritis | M06 |
| Rheumatoid arthritis | M05 |
| Systemic lupus erythematosus (SLE) | M32 |
| Sjögren syndrome | M35.0 |
| Behçet's disease | M35.2 |
| Inflammatory bowel disease | K50-52 |
| Systemic sclerosis | M34 |
| Multiple Sclerosis (MS) | G35 |
| Diseases of liver | K70-K77 |
| Heart failure | I50 |
| Ischemic heart diseases | I20-I25 |
| Cerebrovascular diseases | I60-I69 |
| Chronic obstructive pulmonary disease (COPD) | J44 |
| Herpes zoster | B02 |
| **Medications** |  |
| ANTIINFLAMMATORY AND ANTIRHEUMATIC PRODUCTS, NON-STEROIDS | M01A |
| Hydroxychloroquine | 5521 |
| Methotrexate | 6851 |
| Sulfasalazine | 9524 |
| Leflunomide | 27169 |
| Azathioprine | 1256 |
| **Laboratory tests** |  |
| C reactive protein | 9063 |
| Erythrocyte sedimentation rate | 9066 |
